# Factors associated with the severity and improvement of fatigue in patients with advanced cancer presenting to an outpatient palliative care clinic

**DOI:** 10.1186/1472-684X-11-16

**Published:** 2012-09-17

**Authors:** Sriram Yennu, Diana L Urbauer, Eduardo Bruera

**Affiliations:** 1 Department of Palliative Care and Rehabilitation Medicine, Unit 1414, The University of Texas MD Anderson Cancer Center, 1515 Holcombe Blvd, Houston, TX, USA; 2 Department of Biostatistics, The University of Texas MD Anderson Cancer Center, Houston, TX, USA

**Keywords:** Fatigue, Advanced cancer, Outpatient palliative care, Symptom control

## Abstract

**Background:**

The aim of this study was to determine factors associated with the severity of cancer related fatigue (CRF) and predictors of improvement of CRF at the first follow-up visit in patients with advanced cancer referred to outpatient palliative care clinic (OPC).

**Methods:**

We reviewed the records of consecutive patients with advanced cancer presenting to OPC. Edmonton Symptom Assessment System (ESAS) scores were obtained at the initial and subsequent visits between January 2003 and December 2008. All patients received interdisciplinary care led by palliative medicine specialists following an institutional protocol. Fatigue improvement was defined as a reduction of ≥2 points in ESAS score relative to the baseline. Descriptive statistics were used to summarize patient characterstics. Univariate analyses were performed and only significant variables were included in multivariate regression analysis to determine factors associated with severity and improvement in CRF.

**Results:**

A total of 1778 evaluable patients were analyzed (median age, 59 years; 52% male). The median time between visits was 15 days. Median fatigue scores on the ESAS were 6 at baseline and 5 at follow-up. Severity of all ESAS items and low serum albumin were associated with fatigue at baseline (p < 0.0001). The improvement of fatigue was observed in 586 patients (33%). The hierarchical model showed that fatigue improved over time (b = −0.009; p = 0.0009). low appetite (odds ratio [OR] = 1.09 per point; p = 0.0113) and genitourinary cancer (OR = 1.74 per point; p = 0.0458) were significantly associated with improvement of fatigue.

**Conclusions:**

CRF is strongly associated with physical and emotional symptoms. Genitourinary cancer and low appetite at baseline were associated with successful improvement of fatigue.

## Background

Previous research has shown that patients with advanced cancer develop severe physical and psychosocial symptoms as a result of cancer and treatments [1,2]. Among cancer-related symptoms, cancer-related fatigue (CRF) is the most common chronic and distressing [2], with a frequency of 60-90% [1]. Since CRF is more severe in advanced stages than in early stages of disease, CRF can prevent patients with advanced cancer from receiving effective cancer therapy [2]. The National Comprehensive Cancer Network defines CRF as a distressing, persistent, subjective sense of physical, emotional, and/or cognitive tiredness or exhaustion related to cancer or cancer treatment that is not proportional to recent activity and that interferes with usual functioning [3]. Despite CRF’s prevalence, severity, and effects on quality of life in patients with advanced cancer, available treatment options are limited [4].

Most referrals to outpatient palliative care clinics (OPC) were made late in the trajectory of the disease [5,6]. Thus patients had only one or two follow-up visits due to late referral and logistics of receiving cancer care in a comprehensive center away from home. Hence it is vitally important to obtain prompt control of CRF in a short period of time. However there limited studies regarding factors associated with the severity of fatigue at the initial visit and predictors of improvement in patients with advanced cancer seen in outpatient palliative care clinics at first follow-up visit. Such data would enable researchers to develop treatment strategies to manage CRF in ambulatory advanced cancer patients. The aim of this study was to determine factors associated with the severity of fatigue and predictors of improvement of fatigue at the first follow-up visit in patients with advanced cancer referred to outpatient palliative care clinic.

## Methods

### Patient eligibility and assessments

We reviewed the charts of 2071 consecutive advanced cancer patients (defined as locally advanced or metastatic) who had received care at the outpatient palliative care clinic (OPC) at The University of Texas MD Anderson Cancer Center and had prospectively completed the Edmonton Symptom Assessment System (ESAS) questionnaire including the ESAS fatigue score at the initial visit and subsequent follow-upvisits between January 2003 and December 2008. These patients had at least one follow-up visit after the initial consultation. Only patients who had prospectively completed an ESAS questionnaire at the initial visit and at any follow-up visits within 7–30 days of the initial visit were eligible for inclusion. Of the 2071 patients screened, a total of 1788 met the eligibility criteria. The patient characterstics and symptoms of patients with follow-up and those who did not were compared to determine if the study sample truly represents the OPC advanced cancer population.

To test the impact of palliative consultation on fatigue between the initial visit and the subsequent follow-up visit, we defined improvement of fatigue as a reduction of ≥2 points from the baseline, which is a threshold based on previous quality of life studies [7,8].

### Process of palliative service and interventions performed at the outpatient palliative care clinic

Care for all patients in the palliative care clinic was provided by an interdisciplinary palliative care team led by board-certified palliative care specialists. All 10 specialists work as a team and provide mutual coverage in cases of absence or illness to avoid clinic cancellations, which helps the team maintain a homogeneous approach to assessment, management, and communication with patients and their families. The other team members include a registered nurse trained specifically in palliative care, a pharmacist, a nutritionist, a chaplain, a social worker, and an advanced nurse practitioner trained in palliative care and psychiatry who provides counseling service. Other specialists in services such as wound management, speech therapy, occupational therapy, and physical therapy are consulted when needed.

The care of all patients followed a standardized management plan [9]. Patients and their families were initially assessed by the registered nurse using ESAS, Memorial Delirium Assessment Scale (MDAS), CAGE and constipation and family support questionnaires. The findings were discussed with a palliative care specialist, who then interviewed the patients and their families and performed a physical examination. The physician and nurse asked appropriate members of the interdisciplinary team to participate depending on the individual needs of patients and their families. Most patients referred to the outpatient palliative care clinic receive active cancer treatment including targeted therapy. In all patients fatigue was treated as a multidimensional construct irrespective of whether it was due to disease, cancer treatment or comorbidities. These interventions and care provided by the interdisciplinary team complied with palliative care guidelines established by the National Comprehensive Cancer Network and National Consensus Project and have been outlined elsewhere [10]. Specifically for the management of fatigue, the clinic coordinates a comprehensive assessment and management of all associated cancer-related symptoms such as pain, anorexia, anxiety, depression, sedation, shortness of breath, and sleep disturbance. The clinic also provides expressive supportive counseling and cognitive interventions so that patients and their families understand the treatment goals. Special emphasis is placed on medication review and any plans to discontinue medications associated with fatigue and sedation such as muscle relaxants, benzodiazepines, and antihistamines. In addition, the clinic team emphasizes exercise, light therapy, and methods to maintain and enhance socialization.

### Edmonton symptom assessment system

The ESAS is a simple, validated, and reliable multi-item instrument developed to measure various symptoms in patients with advanced cancer [11,12]. The ESAS questionnaire, visualized graphically as a numerical rating scale (0–10), was used to evaluate nine items (pain, fatigue, nausea, depression, anxiety, drowsiness, appetite, well-being, and shortness of breath) and one patient-specific symptom; the symptom distress score is calculated by adding the scores for the nine ESAS items. The patients were asked to rate the average intensity of these symptoms in the previous 24 hours, with higher scores indicating higher symptom intensity. In the outpatient settings the vast majority of patients are able to complete ESAS by themselves with minimal assistance. The ESAS fatigue score was used to measure changes in fatigue.

### The memorial delirium assessment scale

The MDAS is a structured, 10-item clinician-rated scale, with each item scored as 0–3 and a possible total score of 0–30, designed to quantify the severity of delirium in medically ill patients. This tool was originally tested in a heterogeneous population of cancer patients and patients without cancer. The MDAS has been validated and used for the diagnosis of delirium in cancer patients [13]. Delirium was defined as an MDAS score of ≥7. MDAS is administered routinely in all patients presenting to the OPC as advanced patient are at risk for delirium.

### CAGE questionnaire

The Cut Down, Annoyed, Guilty, and Eye Opener (CAGE) questionnaire is a simple, four-item screening survey for alcoholism. Previous studies by our group showed that patients who scored positively for alcoholism, with positive responses to ≥2 of the four items, had higher symptom expression than those who scored negatively [14]. CAGE questionnaire is administered routinely in all patients presenting to the OPC as advanced patients have severe symptom distress including significant pain and are on opioids and psychostimulants.

We received University of Texas MD Anderson Cancer Center Institutional Review Board approval for the study (protocol DR01-0710).

### Statistical analyses

Descriptive statistics (means, medians, frequencies, and percentages) were used to summarize the patients’ symptoms measured by ESAS, CAGE and MDAS and their demographic characteristics. Comparison of patient characteristics and symptoms of patients with initial consult and follow-up visit and patients with no follow-up visit were compared using *t*-test, and Fisher exact *t* test. Statistical significance was set at p-value ≤0.05. To determine the association between baseline fatigue and gender, race, cancer site, anemia status, albumin level, and alcoholism status, we calculated summary statistics for ESAS for each of these variables and used a Kruskal-Wallis test to determine whether the distribution of baseline fatigue was the same for each. We also calculated correlation coefficients to determine the association between age and other baseline ESAS items.

To determine the predictive factors associated with severity of fatigue at baseline a linear regression model was created with the baseline fatigue score as the dependent variable and all the other baseline ESAS scores, MDAS score, alcoholism, low albumin (<3.5 g/dl anemia (hemoglobin <10 g/dl), and primary cancer type as the independent variables. All variables that were statistically significant in the univariate analyses (p ≤ 0.10) were included in the linear regression model.

Finally, a logistic regression model was created to determine whether ESAS score, MDAS score, anemia, low albumin, alcoholism, or primary cancer type at baseline was related to improvement in fatigue, with improvement of fatigue defined as a decrease of ≥2 in the ESAS fatigue score [7]. In these analyses, a p value of <0.05 was considered significant.

## Results

Table 1 summarizes the demographic and clinical characteristics of the study population. The 1778 evaluable patients had a median age of 59 years; 52% were male. The most common primary cancer types were head and neck cancer and lung cancer (27%). The median time between visits was 15 days. The mean (standard deviation) fatigue score at baseline was 6 (2.39). 1489 patients (80%) reported moderate or severe fatigue (≥4/10).

**Table 1:** Patient Characteristics(n = 1778)

**Characteristics**		**Number**	**Percentage (%)**
Age in years	Mean(SD)	58(13)	
	Median	59	
	Range	15-90	
Gender	Male	918	52%
Race	Caucasians	1213	68%
Cancer type	Lung or Head and Neck	473	27%
	Gastrointestinal	301	17%
	Genitourinary	176	10%
	Sarcoma	68	4%
	Gynecologic	120	7%
	Breast	128	7%
	Other†	512	28%
Severity of baseline fatigue	Mean(SD)	6(2.39)	
	Median(Q1-Q3)	6(4–8)	
	Mild (0–3)	293	16%
	Moderate (4–6)	909	51%
	Severe (7–10)	580	33%
	Moderate or Severe (4–10)	1489	84%

Abbrevations: SD, Standard deviation.

† Leukemias, Lymphomas, unknown primary, melanoma.

Table 2 summarizes the ESAS item scores at the initial presentation and the first follow-up visit. At the initial visit, the median MDAS score was 2 (normal range, 0–7 of a possible 30; n = 793), the median blood hemoglobin level was 11.3 g/dl (normal range, 12–16 g/dl; n = 1426), and the median serum albumin level was 3.7 g/dl (normal range, 3.5-4.7 g/dl; n = 1178). Of the 793 patients with MDAS data, 31 (1.74%) had delirium according to MDAS (≥7 of 30). Of 1426 patients, 314 (17.6%) had anemia (≤10 g/dl), and of the 1178 patients with albumin data, 192 (10.7%) had low albumin levels (<3.5 g/dl).

**Table 2:** ESAS scores at the initial presentation and at the first follow-up visit

**ESAS item**	**Initial visit† (n = 1778)**	**1st Follow-up visit† (n = 1778)**
Pain	5(3–8)	4(2–7)
Fatigue	**6(4–8)**	**5(3–8)**
Nausea	1(0–4)	0(0–3)
Depression	3(0–5)	2(0–4)
Anxiety	3(0–5)	2(0–5)
Appetite	4(1–6)	3(1–6)
Drowsiness	5(2–7)	4(1–6)
Feeling of Well-being	5(3–7)	4(2–6)
Shortness of breath	2(0–5)	2(0–4)
Sleep disturbance	5(2–7)	4(2–6)
ESAS Symptom distress score	34(24–46)	30(20–41)

ESAS = Edmonton symptom assessment scale.

†Median (25^th^-75^th^ quartiles).

Table 3 shows baseline data for 1788 patients who met the eligibility criteria and for 283 who had no follow-up and therefore were not evaluable. The results show that the patients who were not evaluable had similar characteristics to those evaluated but had lower severity of ESAS scores.

**Table 3:** Comparison of Patient Characteristics Between Outpatient Palliative Clinic Patients with and without follow-up

	**Patients with follow-up (n = 1778)**		**Patients with no follow-up (n = 283)**	**p-value†**
**Characteristics**		**Number(%)**		
Age	Mean(SD)	58(13.4)	59(13.4)	0.50
	Median	59	60	
Gender	Male	918(52%)	130(46%)	0.10
Cancer type	Lung and Head&Neck	473(27%)	104(37%)	0.50
	Gastrointestinal	301(17%)	46(16%)	
	Genitourinary	176(10%)	43(15%)	
	Sarcoma	68(4%)	11(4%)	
	Gynecologic	120(7%)	24(8%)	
	Breast	128(7%)	17(6%)	
	Others	426(24%)	38(14%)	
ESAS items	Median(Qtl 25^th^-75^th^)		Median(Qtl 25^th^-75^th^)	
Pain	5(3–8)		3(1–5)	<0.001
Fatigue	6(4–8)		3(0–3)	<0.001
Nausea	1(0–4)		3(0–3)	<0.001
Depression	3(0–5)		0(0–1)	<0.001
Anxiety	3(0–5)		0(0–3)	<0.001
Appetite	4(1–6)		2(0–4)	<0.001
Drowsiness	5(2–7)		0(0–3)	<0.001
Feeling of well-being	5(3–7)		2(0–4)	<0.001
Shortness of Breath	2(0–5)		0(0–3)	<0.001
Sleep disturbance	5(2–7)		2(0–4)	<0.001
ESAS-Symptom distress score	34(24–46)		28(17–40)	<0.001

ESAS = Edmonton Symptom Assessment Scale.

† *t*-test for age and ESAS; Fisher exact test for other variables.

We found no univariate associations between fatigue and age (p = 0.06), gender (p = 0.07), race (p = 0.11), type of cancer (p = 0.32), anemia (p = 0.09) or alcoholism (p = 0.18) (Table 4). There were correlations between fatigue and severity levels of pain (r = 0.23), nausea (r = 0.31), anxiety (r = 0.33), depression (r = 0.33), drowsiness (r = 0.29), appetite (r = 0.41), sleep disturbance (r = 0.25), shortness of breath (r = 0.33), and well-being (r = 0.36) and the total ESAS symptom distress score (r = 0.54), with p < 0.0001 for each. According to the linear regression model, factors associated with fatigue were levels of pain (p < 0.0001), depression (p = 0.0017), appetite (p < 0.0001), drowsiness (p < 0.0001), well-being (p < 0.0001), shortness of breath (p < 0.0001), albumin level (p < 0.0001), and nausea status (p = 0.0001) (Table 5). In this predictive model, adjusted r^2^ = 0.33.

**Table 4:** Severity of Baseline Fatigue by Patient Characterstics

	**N**	**Mean (SD)**	**Median**	**p-value**
**Gender**				0.07
Female	859	6.16 (2.4)	6	
Male	918	5.97 (2.38)	6	
**Race**				0.11
White	1009	6.22 (2.27)	6	
Black	155	6.36 (2.44)	7	
Hispanic	142	5.86 (2.13)	6	
Asian	48	5.83 (2.68)	6	
**Cancer Site**				0.32
Breast	93	6.48 (2.2)	7	
Gastrointestinal	280	6.23 (2.36)	6	
Genitourinary	162	5.99 (2.36)	6	
Gynecological	120	6.43 (2.44)	7	
Head and Neck, Lung	472	6.12 (2.23)	6	
Sarcoma	68	5.79 (2.34)	5.5	
Other	176	6.18 (2.32)	6	
**Anemia**				0.09
** No**	1112(83.4%)	6.02 (2.39)	6	
** Yes**	314(10.7%)	6.26 (2.43)	7	
**Low Albumin**				**0.0003**
** No**	986(89.3%)	6 (2.43)	6	
** Yes**	192(10.7%)	6.69 (2.33)	7	
**Alcoholic**				0.18
** No**	1044(85%)	6.09 (2.43)	6	
** Yes**	184(15%)	6.34 (2.31)	7	

**Table 5:** Independent Predictors of the severity of Baseline fatigue

	**Full model**		**Reduced model**	
**Variable**	**B (SE)**	**p-value**	**B (SE)**	**p-value**
**ESAS items**				
**Pain**	0.09 (0.02)	**< 0.0001**	0.09 (0.02)	**< 0.0001**
**Nausea**	0.09 (0.02)	**0.0001**	0.09 (0.02)	**0.0001**
**Depression**	0.09 (0.03)	**0.0017**	0.1 (0.02)	**< 0.0001**
**Anxiety**	0.03 (0.03)	0.31		
**Appetite**	0.17 (0.02)	**< 0.0001**	0.17 (0.02)	**< 0.0001**
**Drowsiness**	0.08 (0.02)	**< 0.0001**	0.08 (0.02)	**< 0.0001**
**Feeling of well-being**	0.12 (0.03)	**< 0.0001**	0.12 (0.03)	**< 0.0001**
**Shortness of breath**	0.14 (0.02)	**< 0.0001**	0.14 (0.02)	**< 0.0001**
**Sleep disturbance**	0.00 (0.02)	0.96		
**Low Albumin**	0.64 (0.16)	**< 0.0001**	0.66 (0.16)	**< 0.0001**
**Male**	−0.10 (0.12)	0.37		
**Anemia**	0.10 (0.15)	0.52		

ESAS- Edmonton symptom assessment scale; B- Beta; SE- standard error.

The hierarchical model showed that fatigue did improve at the first follow-up visit (b = −0.009, p = 0.0009), and the logistic regression model showed that low appetite, genitourinary (GU) cancer, high nausea, and low shortness of breath were predictors of this improvement of fatigue by outpatient palliative care consultation. Baseline low appetite was the strongest predictor of improvement in the reduced model (odds ratio [OR] = 1.09 per point; p = 0.0113). Additionally, patients with GU cancer were more likely to improve in fatigue in the reduced model (OR = 1.74 per point; p = 0.0458) (Table 6). The improvement of fatigue was observed in 586 patients (33%). Baseline nausea was the strongest predictor of improvement in fatigue with higher baseline nausea scores predictive of improvement (OR = 1.07 per point; p = 0.0442). In patients with severe fatigue (score 8–10), those with less shortness of breath at baseline were more likely to improve in fatigue (OR = 0.88 per point, p = 0.0068).

**Table 6:** Factors associated with improvement of fatigue

**Variable**	**Full model**^**a**^			**Reduced model**		
**ESAS items**	**OR**	**p-value**	**95% CI**	**OR**	**p-value**	**95% CI**
**Pain**	1.05	0.18	0.98 - 1.12	1.05	0.12	0.99 - 1.12
**Nausea**	1.03	0.51	0.95 - 1.11			
**Depression**	1.02	0.63	0.94 - 1.11			
**Anxiety**	0.98	0.63	0.90 - 1.07			
**Appetite**	1.08	0.01	1.01 - 1.16	1.09	**0.01**	1.02 - 1.16
**Drowsiness**	0.95	0.12	0.88 - 1.02	0.96	0.16	0.90 - 1.02
**Feeling of well-being**	1.09	0.06	1.00 - 1.19	1.08	0.06	1.00 - 1.17
**Shortness of breath**	0.97	0.45	0.91 - 1.04			
**Sleep disturbance**	1.00	0.93	0.93 - 1.08			
**Anemia**	0.86	0.55	0.52 - 1.43			
**Low albumin**	1.25	0.50	0.66 - 2.37			
**MDAS items**						
**Consciousness**	1.41	0.42	0.61 - 3.28			
**Disorientation**	0.89	0.65	0.55 - 1.46			
**Memory change**	0.78	0.24	0.52 - 1.18			
**Digit Span**	0.71	0.27	0.38 - 1.32	0.69	0.16	0.41 - 1.17
**Attention**	0.55	0.07	0.28 - 1.07	0.57	0.07	0.31 - 1.05
**Disorganized thinking**	1.26	0.21	0.87 - 1.81			
**Perception distrurbance**	0.68	0.23	0.35 - 1.29			
**Delusions**	1.20	0.56	0.63 - 2.29			
**Psychomotor activity**	1.34	0.58	0.47 - 3.86			
**Sleep disturbance**	1.06	0.76	0.73 - 1.54			
**Alcoholism**	0.82	0.50	0.46 – 1.46			
**Cancer Types**^**b**^						
**Breast**	0.84	0.66	0.38 – 1.85			
**Gastrointestinal**	1.07	0.80	0.63 - 1.81			
**Genitourinary**	1.92	0.04	1.02 - 3.62	1.74	**0.04**	1.01 - 2.99
**Gynecologic**	1.19	0.65	0.56 - 2.53			
**Sarcoma**	1.34	0.47	0.60 - 3.01			
**Others**^**c**^	1.17	0.60	0.65 - 2.11			

ESAS: Edmonton symptom assessment scale; MDAS: Memorial delirium assessment scale;

^a^- Logistic regression model.

^b^- Reference: Head & Neck or Lung cancer.

^c^- Unknown primary, melanoma, skin, leukemia, lymphoma, brain.

Changes in fatigue were positively associated with changes in the other cancer related symptoms as assessed by ESAS (Table 7).

**Table 7:** Association of change in Fatigue with change in other ESAS symptoms

**ESAS symptoms**	**r**	**P-value**
**Pain**	.294	<0.001
**Nausea**	.296	<0.001
**Depression**	.288	<0.001
**Anxiety**	.277	<0.001
**Appetite**	.419	<0.001
**Drowsiness**	.292	<0.001
**Well being**	.354	<0.001
**Shortness of Breath**	.285	<0.001
**Sleep**	.259	<0.001

ESAS: Edmonton Symptom Assessment Scale.

r: Correlation coefficient.

## Discussion

Our findings provide preliminary evidence that palliative care consultation for fatigue for advanced cancer patients seen at an outpatient palliative care clinic in a comprehensive cancer center was associated with improvement of fatigue at the time of the first follow-up visit.

Moderate to severe fatigue was common (84%) in the patients we evaluated. Severity of fatigue at the time of consultation significantly correlated with all ESAS items, with pain and appetite having the strongest associations. These findings are consistent with those from previous studies [15-23].

One of the strengths of this study, despite its retrospective design, is that the fatigue assessment was performed prospectively at both the initial and subsequent follow-up visits using validated tools in a dedicated outpatient palliative care clinic. The fatigue assessment was completed by the patients under the supervision of nurses trained specifically in palliative care, and standardized management was provided by a specialist-led palliative care team in accordance with our institutional protocol based on the findings from previous fatigue assessments [9]. Moreover, this study differed from earlier studies in its relatively large population sample size of over 1700 patients and its focus on factors associated with improvement in fatigue after an outpatient palliative care consultation. Thus, our findings provide preliminary reference data about the effectiveness of outpatient palliative care when standard palliative care is applied to patients with fatigue.

We found that the severity levels of pain, depression, appetite, nausea, drowsiness, well-being, and shortness of breath and albumin level were predictive of the severity of fatigue at the time of the initial consultation. This information emphasizes the importance of thorough serial assessment of all cancer-related symptoms for optimal management of fatigue. Future prospective trials are needed, and fatigue interventions should incorporate a multimodal interdisciplinary approach with targets including the treatment of fatigue-related symptoms such as pain, low appetite, and depression in addition to specific pharmacological interventions for fatigue. These results are consistent with prior studies by Hwang et al [20], who found that shortness of breath, pain, lack of appetite, drowsiness, sadness, and irritability predicted fatigue. However, female gender and low hemoglobin levels, which prior studies have found to be predictive of severity of fatigue [24-26], were not associated with fatigue in our study. This result could be due to the overwhelmingly high symptom burden in our patient population, which may reduce the role of factors such as anemia [27-29].

We also found that severe anorexia at the initial visit to the palliative care clinic was associated with improvement of fatigue. This may be due to a strong association of fatigue with anorexia and cachexia, as both of which may be caused by the same pathophysiologic process, namely inflammation [30-32].

We also found that patients with GU cancers (prostate, renal, and transitional cell cancers) were more likely to improve in fatigue (OR = 1.74 per point, p = 0.045). In patients with prostate cancer, anorexia cachexia is less important contributor to fatigue [31]. The authors speculate that fatigue as a result of androgen deprivation in prostate cancer patients may be more amenable to palliative interventions such as exercise [33,34]. However, further studies are needed to understand this result.

The results of his study suggest that the changes in the fatigue scores were positively associated with the changes in the severity of other ESAS symptoms; these finding would strongly imply a causal relation between symptom load and fatigue.

Though the results of this study show preliminary evidence that palliative care consultation was successful in reducing the severity of fatigue, our model did not capture all the factors associated with improvement of fatigue, as indicated by adjusted r^2^ of 0.33. Further studies are needed.

The results show that the severity of fatigue and other ESAS symptoms in patients who did not have a follow-up visit (excluded patients) were milder than in those who had at least one follow-up visit (the study sample) (Table 3). This analysis validates that the results are a good representation of the patients seen in outpatient palliative care.

Our study has several limitations, the most important of which was the retrospective design and its lack of patients in the setting other than an outpatient palliative care clinic. Another limitation is the use of single item measure to assess physical and emotional symptoms using ESAS. However prior studies have shown that ESAS items and other single item questionnaires correlate well with multi-item symptom assessment tools [22,35-38]. Although in the vast majority cases patient complete the ESAS by themselves in outpatient setting there is a possibility that in the most fatigued patients the nurse or caregiver could have introduced the bias by assisting the patient. More research is necessary to address this possibility. This study also did not assess other well-known factors that may contribute to fatigue such as inflammatory biomarkers, which play an important role in fatigue causation [32,39,40]. Some of the statistically significant associations in this study may be as a result of multiple analysis. Future prospective studies are needed to confirm these findings.

## Conclusion

Our findings suggest that fatigue is the most severe symptom in patients with advanced cancer. Severity levels of pain, depression, appetite, nausea, drowsiness, well-being, and shortness of breath and the albumin level were predictive of the severity of fatigue at the time of the initial consultation, with pain and low appetite being the most significant predictors. Genitourinary cancer and low appetite at baseline were associated with successful improvement of fatigue.

## Competing interests

The author(s) declare that they have no competing interests.

## Authors’ contributions

SY and EB were involved in the study concept, design, assembly, analysis, interpretation of the data and drafting the manuscript. DLU was involved in the design, assembly, analysis, interpretation of the data and drafting of the manuscript. All authors read and approved the final manuscript.

## Pre-publication history

The pre-publication history for this paper can be accessed here:

http://www.biomedcentral.com/1472-684X/11/16/prepub
